# Genetic signatures shared in embryonic liver development and liver cancer define prognostically relevant subgroups in HCC

**DOI:** 10.1186/1476-4598-11-55

**Published:** 2012-08-14

**Authors:** Diana Becker, Ioannis Sfakianakis, Markus Krupp, Frank Staib, Aslihan Gerhold-Ay, Anja Victor, Harald Binder, Maria Blettner, Thorsten Maass, Snorri Thorgeirsson, Peter R Galle, Andreas Teufel

**Affiliations:** 1 Department of Medicine I, Johannes Gutenberg University, Mainz, Germany; 2 Institute of Medical Biostatistics, Epidemiology and Informatics(IMBEI), Johannes Gutenberg University, Mainz, Germany; 3 Laboratory of Experimental Carcinogenesis (LEC), National Cancer Institute, National Institutes of Health, Bethesda, MD, USA; 4 Department of Medicine I, Johannes Gutenberg University, Building 605, Langenbeckstr. 1, 55101, Mainz, Germany

## Abstract

Multiple activations of individual genes during embryonic liver and HCC development have repeatedly prompted speculations about conserved embryonic signatures driving cancer development. Recently, the emerging discussion on cancer stem cells and the appreciation that generally tumors may develop from progenitor cells of diverse stages of cellular differentiation has shed increasing light on the overlapping genetic signatures between embryonic liver development and HCC. However there is still a lack of systematic studies investigating this area. We therefore performed a comprehensive analysis of differentially regulated genetic signaling pathways in embryonic and liver cancer development and investigated their biological relevance.

Genetic signaling pathways were investigated on several publically available genome wide microarray experiments on liver development and HCC. Differentially expressed genes were investigated for pathway enrichment or underrepresentation compared to KEGG annotated pathways by Fisher exact evaluation. The comparative analysis of enrichment and under representation of differentially regulated genes in liver development and HCC demonstrated a significant overlap between multiple pathways. Most strikingly we demonstrated a significant overlap not only in pathways expected to be relevant to both conditions such as cell cycle or apoptosis but also metabolic pathways associated with carbohydrate and lipid metabolism. Furthermore, we demonstrated the clinical significance of these findings as unsupervised clustering of HCC patients on the basis of these metabolic pathways displayed significant differences in survival.

These results indicate that liver development and liver cancer share similar alterations in multiple genetic signaling pathways. Several pathways with markedly similar patterns of enrichment or underrepresentation of various regulated genes between liver development and HCC are of prognostic relevance in HCC. In particular, the metabolic pathways were identified as novel prognostically relevant players in HCC development.

## Introduction

Hepatocellular carcinoma (HCC) is the fifth most common cancer worldwide and its incidence is rising [1,2]. In contrast to other cancers, therapeutic options other than surgery remain very limited, and it was only three years ago that a drug, sorafenib, first showed a benefit in patient survival [3] Thus, exploring the genetic mechanisms leading to HCC development warrants being further evaluated, especially with respect to the identification of novel drug targets.

It has repeatedly been reported that several genes are relevant to both embryonic liver development and liver cancer. Recently, several studies on liver embryonic development have established the concept that the genetic programs controlling liver development are often deregulated in liver cancer. Signaling transductory pathways including Wnt-signaling pathway [4-9], TGF-β signaling pathway [10-12], MAPK signaling pathway [13,14], Jak-STAT signaling pathway [15,16], Notch signaling pathway [17,18], and the Hedgehog signaling pathway [19,20] have been reported to play important roles in hepatoblast proliferation and differentiation during embryonic development, as well as in hepatocarcinogenesis. Since many biological mechanisms such as cell cycle control, growth and proliferation are essential to both embryonic development and cancer de-differentiation, this may not be completely surprising. A pioneer study analysed a comprehensive microarray data set of mouse liver development during multiple stages. Li et al. reported that genes enhanced in early stages of liver development are also enriched in HCC development [21-23].

There has been renewed interest in these observations over recent years as they would be in accordance with a cancer stem cell hypothesis for hepatocellular carcinoma. Although such a stem cell hypothesis is still a matter of debate, it has been repeatedly documented that solid tumors contain a small subgroup of tumorigenic cells which can generate new tumors in xenograft transplantation [24]. This subpopulation was termed cancer stem cells since they possess stem cell-like properties and contribute to a hierarchical structure containing varied progenies [25]. Simultaneously, fetal liver-derived hepatic stem/progenitor cells over expressing Bmi1 or mutant B-catenin may acquire enhanced self-renewal capability and tumorigenicity to initiate HCC [26]. Finally, unsupervised clustering of patients with HCC based on their gene expression profiles show that patients with HCC and gene expression profiles similar to hepatic stem/progenitor cells had a poorer prognosis [27,28]. Together, it is clear that liver carcinogenesis shares common genetic programs with liver development. However, systematic analysis of genetic signaling pathways or genetic signatures essential to both embryonic development and cancer had not previously been performed.

Thus, studying liver development may not only be valuable from an embryologic perspective but also to contribute to a better understanding of the pathogenesis of liver cancer. Deciphering common molecular events may be useful for unraveling regulatory mechanisms which can impact on cancer diagnosis and treatment, and separate them from regulated but non-essential bystander genes.

In the absence of a systematic, genome wide comparison of genetic expression profiles and genetic pathways, we performed such an analysis based on the pathway annotation of the Kyoto Encyclopedia of Genes and Genomes (KEGG) database [29,30] curated pathways (http: http://www.genome.jp/kegg/pathway.html). Furthermore, we evaluated enriched or underrepresented pathways for their relevance with respect to prognosis of patients with HCC.

## Results

### Enriched signaling pathways in mouse liver development

Multiple genes have been identified to be differentially regulated during liver development [21-23]. However, only scant data is available on the interaction of these genes. Since we had earlier pointed out that liver development is unlikely to be due to only a few individual master regulators, one has to assume a complex interaction of genetic networks drive liver development [31]. We have therefore analyzed the regulation of signaling pathways in liver development using microarray datasets from embryonic mouse liver development over multiple time points (GSE13149 and GSE11201), microarray datasets of two murine HCC models (GSE8642 and GSE9012), and a human HCC datatset containing micrarray data from 139 HCC patients [27,31]. KEGG contained a total of 258 pathways. Of these, 174 (67%) pathways were deregulated in at least one developmental stage. The pathway category “signaling molecules and interaction” emerged to be the most frequently altered cellular biological process in liver development, as it was deregulated in 80% (61/76) of all investigated embryonic stages (p-value ranged between 3.5e-32 and 0.04).

Furthermore, genes of the “ECM-receptor interaction pathway” were enriched among genes differentially regulated during liver development and most of these genes were upregulated with highest enrichment of up-regulated genes in perinatal and postnatal stages (p-value ranged between 4.6e-05 and 0.04).

“Cell growth and death” related signaling pathways were deregulated in 57% (43/76) of investigated stages and developmental arrays, with the subcategories “cell cycle” and “p53 signaling pathway” to be enriched in differentially regulated genes during liver development with up-regulation of “cell cycle” in embryonic and perinatal stages and “p53 signaling pathway” in almost all stages of liver development (p-value ranged between 5.6e-38 and 3.4e-07, and between 9.8e-07 and 0.02, for cell cycle and p53 signaling pathway, respectively).

Major deregulations were observed in metabolic signaling pathways. Among those, “nucleic acid metabolism” was the most often altered metabolic process during liver development, as it was deregulated in 84% (32/38, p-values ranging between 7.0e-09 and 0.02) over all time points and developmental arrays followed by “lipid metabolism” and “carbohydrate metabolism”. “Lipid metabolism” and “carbohydrate metabolism” were deregulated in 63% (192/304, p-values ranging between 4.4e-10 and 0.05) and 54% (164/304, p-values ranging between 9.5e-08 and 0.05) of all developmental stages, respectively. In contrast to the former pathways showing enrichment during liver development, most genes of these metabolic signatures were down-regulated. Down-regulation of many lipid-related pathways including androgen and estrogen metabolism (p-value ranged between 0.0001 and 0.05), fatty acid metabolism (p-value ranged between 1.2e-08 and 0.04), bile acid biosynthesis (p-value ranged between 1.7e-06 and 0.03) and carbohydrate-related pathways including ascorbate and aldarate metabolism (p-value ranged between 5.9e-07 and 0.02), pentose and glucuronate interconversions (p-value ranged between 2.4e-06 and 0.009) was observed in almost all stages during liver development among pathways with the highest enrichment in down-regulated genes in almost all developmental stages.

### Enriched signaling pathways in Hepatocellular carcinoma

Out of the 258 KEGG curated pathways, 119 were deregulated in at least one liver cancer array. Since the Lee [27,32] dataset deminstrated at least two subgroups of patients we investigated the enrichment of signalling pathways also for genes deregulated only in 20% (Lee20) or 50% (Lee50) of patients. The pathway category “signaling molecules and interaction” was the most affected biological process in HCC, as it was deregulated in 75% (Lee20: 9/12) or 50% (Lee50: 6/12), with the “ECM-receptor interaction” pathway being the most frequently up-regulated pathway in HCC. It was up-regulated in 100% (Lee20: 3/3, Lee50: 3/3) malignant cancer arrays in both human and mice (p-value ranged between 1.0e-05 and 0.02).

Furthermore, the pathway category “cell growth and death” was deregulated in 44% (Lee20: 4/9) and 22% (Lee50: 2/9) of malignant cancer arrays with “cell cycle” being the most frequently altered pathway of this KEGG subcategory as it was enriched in differentially regulated genes in 67% (lee20: 2/3) and 33% (Lee50: 1/3) malignant cancer arrays (p-value ranged between 3.8e-05 and 0.03) followed by “p53 signaling pathway” (p-value equal to 0.003).

Metabolic pathways were also identified to be altered in HCC. The pathway category “lipid metabolism” was the most affected metabolic process in HCC, as it was deregulated in 46% (Lee20: 22/48) and 33% (Lee50: 16/48) of malignant cancer arrays followed by “carbohydrate metabolism” category, deregulated in 38% (Lee20: 18/48) and 33% (Lee50: 16/48). Down-regulation of affected genes was the major trend of regulation in these metabolic processes. “Linoleic acid metabolism” emerged the most frequently deregulated lipid-related metabolic pathway, as it was deregulated in all liver cancer arrays (p-value ranged between 2.4e-06 and 0.03) followed by “fatty acid metabolism” (p-value ranged between 1.1e-10 and 0.03), “androgen and estrogen metabolism” (p-value ranged between 0.0002 and 0.002), and “bile acid biosynthesis” (p-value ranged between 4.5e-06 and 0.01). For the carbohydrate-related metabolic pathways, “pentose and glucuronate interconversions” appeared to be the most frequently deregulated pathways, as it was deregulated in 100% (Lee20: 3/3, Lee50: 3/3) malignant cancer arrays (p-value ranged between 2.2e-05 and 0.02) followed by “ascorbate and aldarate metabolism” (p-value ranged between 0.0004 and 0.02), and “butanoate metabolism” (p-value ranged between 2.2e-08 and 0.03).

These results were further validated in a second, human HCC dataset (Additional file 1: Table S7) [33]. We confirmed the pathway category “signaling molecules and interaction” to be the most enriched biological process in human HCC. Furthermore, we also confirmed a significant enrichment of established cancerogenic pathways such as “Cell cycle”, “p53 signaling pathway”, or “ECM-receptor interaction”. Besides these already established cancer related pathways, we again demonstrated an enrichment of signaling pathways related to lipid and carbohydrate metabolism.

### Commonly enriched signaling pathways in hepatocellular carcinoma and liver development

Investigating genetic signaling pathways in both embryonic liver and HCC development, we were able to demonstrate that it is not only single genes that may be differentially regulated in both conditions but also that there is a significant overlap between enriched signaling pathways in liver development and HCC. Of the 258 pathways listed in the KEGG database, a total of 112 (43%) pathways were deregulated in at least one malignant cancer array and one developmental array. Most of the pathways already reported above to be enriched in either liver or HCC development were also overlapping in enrichment between these two conditions.

**Figure 1:**
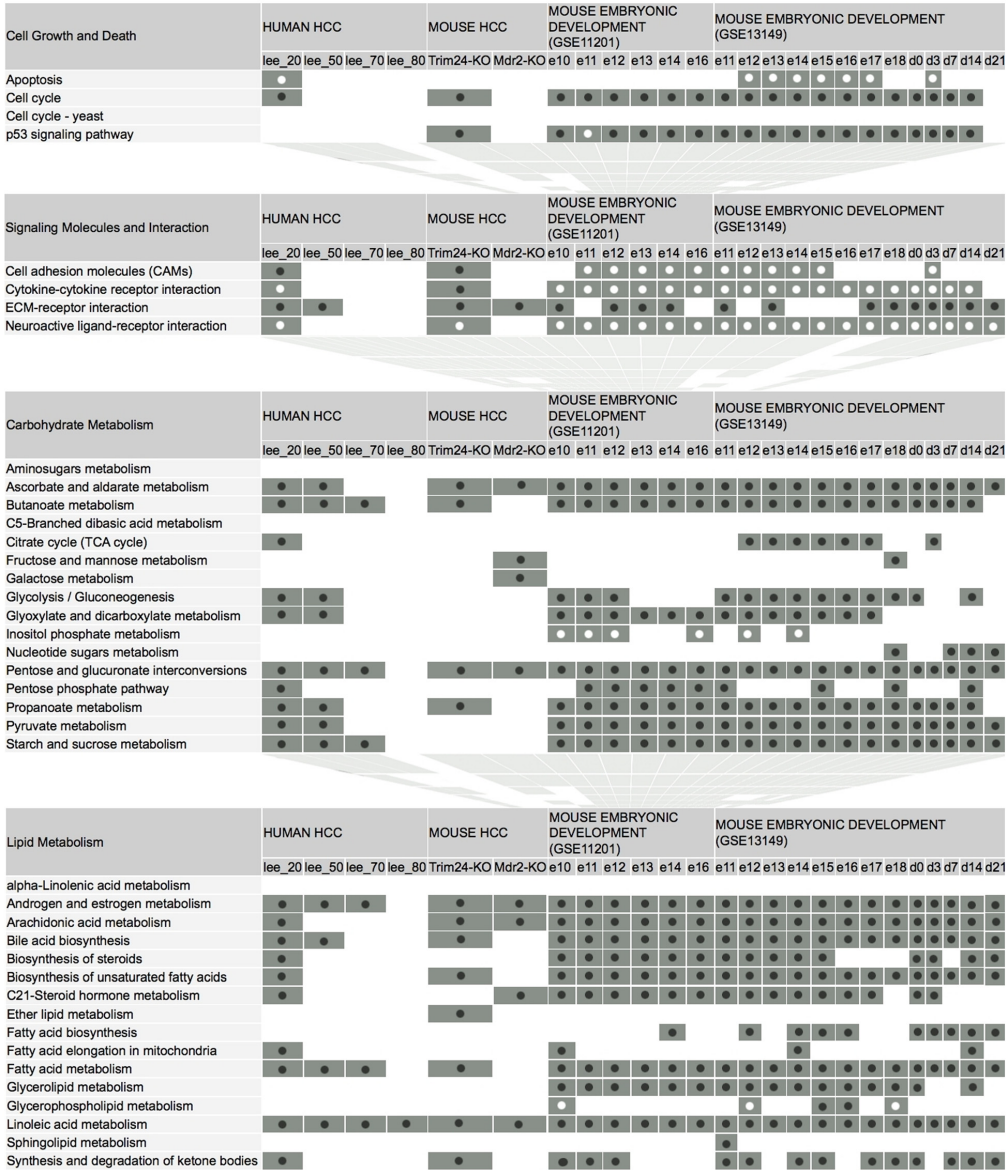
**Summary of signaling pathways with highest overlap of enrichment of differentially regulated genes during embryonic liver and HCC development.** Each grey square at the grid intersection between pathway and developmental stage represents a significant enrichment (black circuits) or under re-presentation (white circuits) of differentially regulated genes of this pathway in the analyzed data set.

The pathway category of “signaling molecules and interaction” was the most enriched cellular process in liver development and liver cancer, as it was deregulated in 75% (Lee20: 9/12) and 50% (Lee50: 6/12) of malignant cancer arrays and in 80.3% (61/76) over 19 developmental arrays across diverse developmental stages. As demonstrated in Figure 1, the overlap / similarity with respect to these pathways in liver development and HCC development was obvious.

Furthermore, we not only demonstrated an enrichment of signaling pathways related to ECM-receptor but even more we were able to demonstrate that the interaction of these mechanisms were highly conserved in malignant cancer arrays as well as in embryonic developmental arrays. The ECM-receptor interaction pathway was deregulated in 100% (Lee20: 3/3, Lee50: 3/3) malignant cancer arrays and in 68,4% (13/19) developmental arrays, suggesting a high relevance of the mechanisms that govern extracellular matrix-transmembrane molecule interactions in both liver cancer and liver development.

Next, several signaling pathways which can be summarized mainly as being essential to lipid metabolism and carbohydrate metabolism were the most affected metabolic processes in HCC, as they were deregulated in 46% (Lee20: 22/48) or 33% (Lee50: 16/48) and 38% (Lee20: 18/48) or 33% (Lee50: 16/48) of malignant cancer arrays, respectively. Following nucleotide metabolism they were the most significantly altered metabolic processes in liver development with lipid metabolism being deregulated in 63% (192/304) of investigated developmental arrays and carbohydrate metabolism in 54% (164/304) of developmental arrays. Down-regulation of multiple genes in many lipid-related pathways could not only be detected by means of superimposed categories of signaling pathways but also on the basis of individual genetic signaling pathways such as “androgen and estrogen metabolism”, “bile acid biosynthesis”, “fatty acid metabolism”, and “linoleic acid metabolism” as well as down-regulation of carbohydrate-related pathways such as “ascorbate and aldarate metabolism”, “pentose and glucuronate interconversions”, and “propanoate metabolism” observed to be highly conserved in malignant cancer arrays and in almost all developmental stages and arrays.

Finally, the pathway category of “cell growth and death” was seen to be altered in developmental arrays with “cell cycle”, and “p53 signaling” pathway, being deregulated in almost all stages during liver development, while in malignant cancer arrays “cell cycle” being the most frequently deregulated pathway of this signaling pathway category. It was deregulated in 67% (Lee20: 2/3) or 33% (Lee50: 1/3) of liver cancer arrays. Intersection of Genes within Pathways between human HCC Liver samples and mouse samples is shown in Additional file 2: Tables S1-S4 and P-value and observed/expected ratio range for each Pathway in Additional file 2: Table S5.

### Survival / Biological relevance

**Figure 2:**
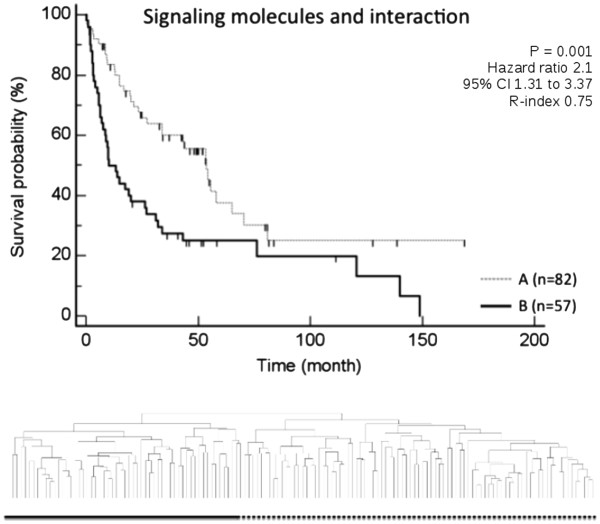
**Kaplan Meier estimated survival for the subgroups identified by unsupervised clustering of 139 patients with HCC based on the pathway category “signaling molecules and interaction”.** Clustering resulted in two prognostically distinct groups (group A contained 82 patients and group B contained 57 patients; p-values were determined by log-rank test).

**Figure 3:**
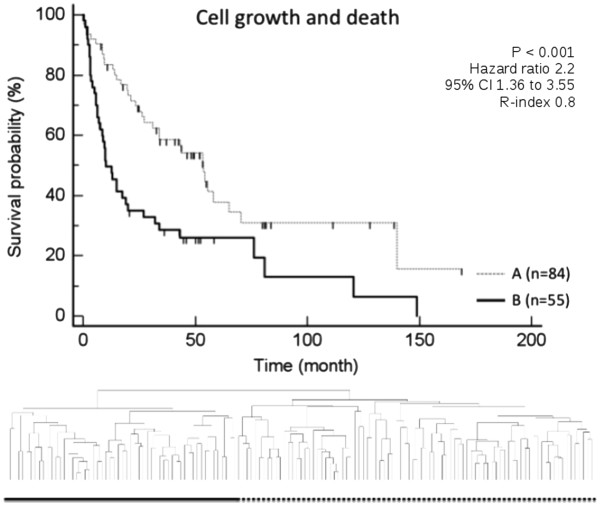
Figure shows Kaplan Meier estimated survival for the subgroups identified by unsupervised clustering of 139 patients with HCC based on the pathway category “cell growth and death” demonstrated two prognostically distinct groups (group A contained 84 patients and group B contained 55 patients; p-values were determined by log-rank test).

**Figure 4:**
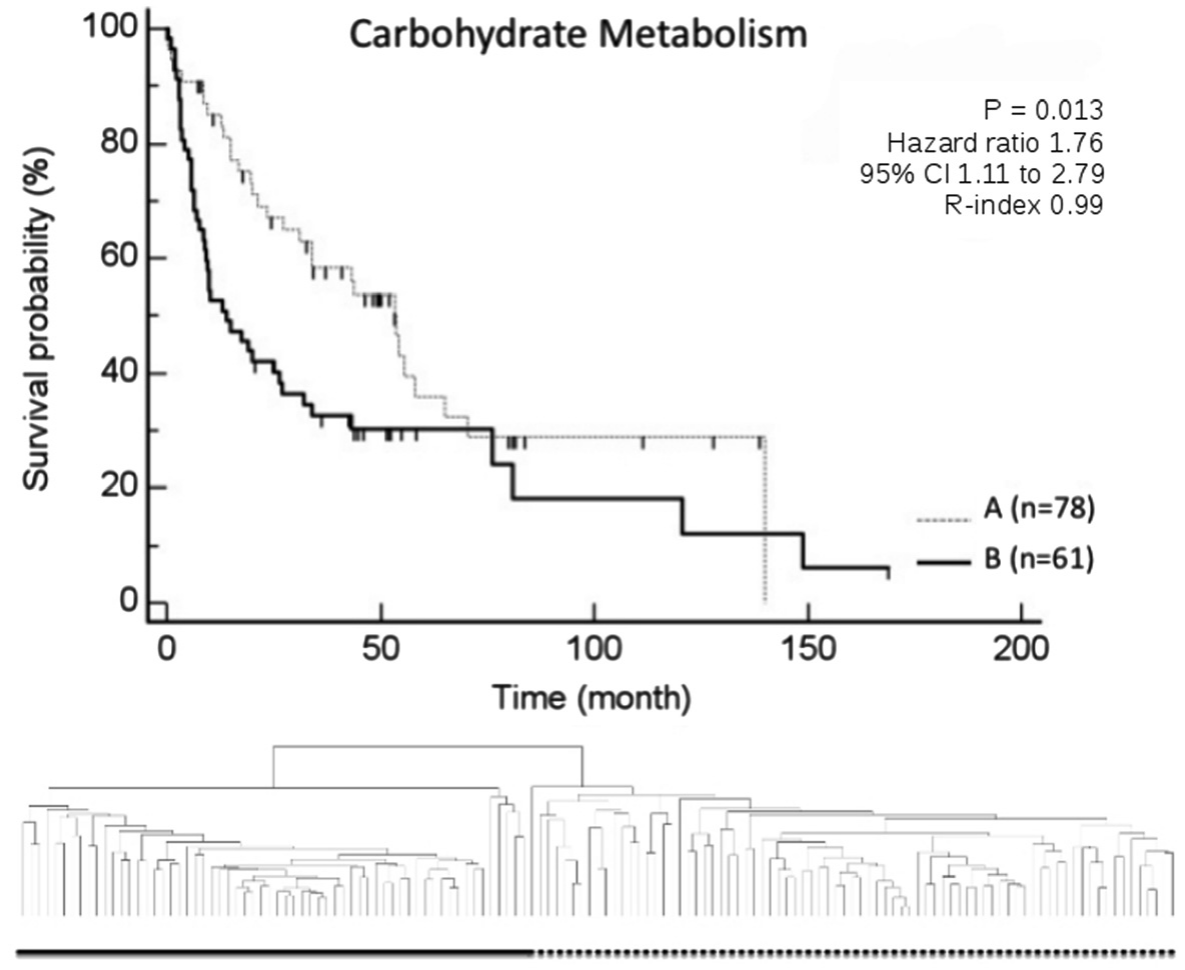
**Based on the expression profile of genes associated with the carbohydrate metabolism, Kaplan Meier estimated survival resulted in two prognostically distinct groups.** These subgroups were identified by unsupervised clustering of 139 patients with HCC based on the pathway category “carbohydrate metabolism” (group A contained 78 patients and group B contained 61 patients; p-values were determined by log-rank test).

**Figure 5:**
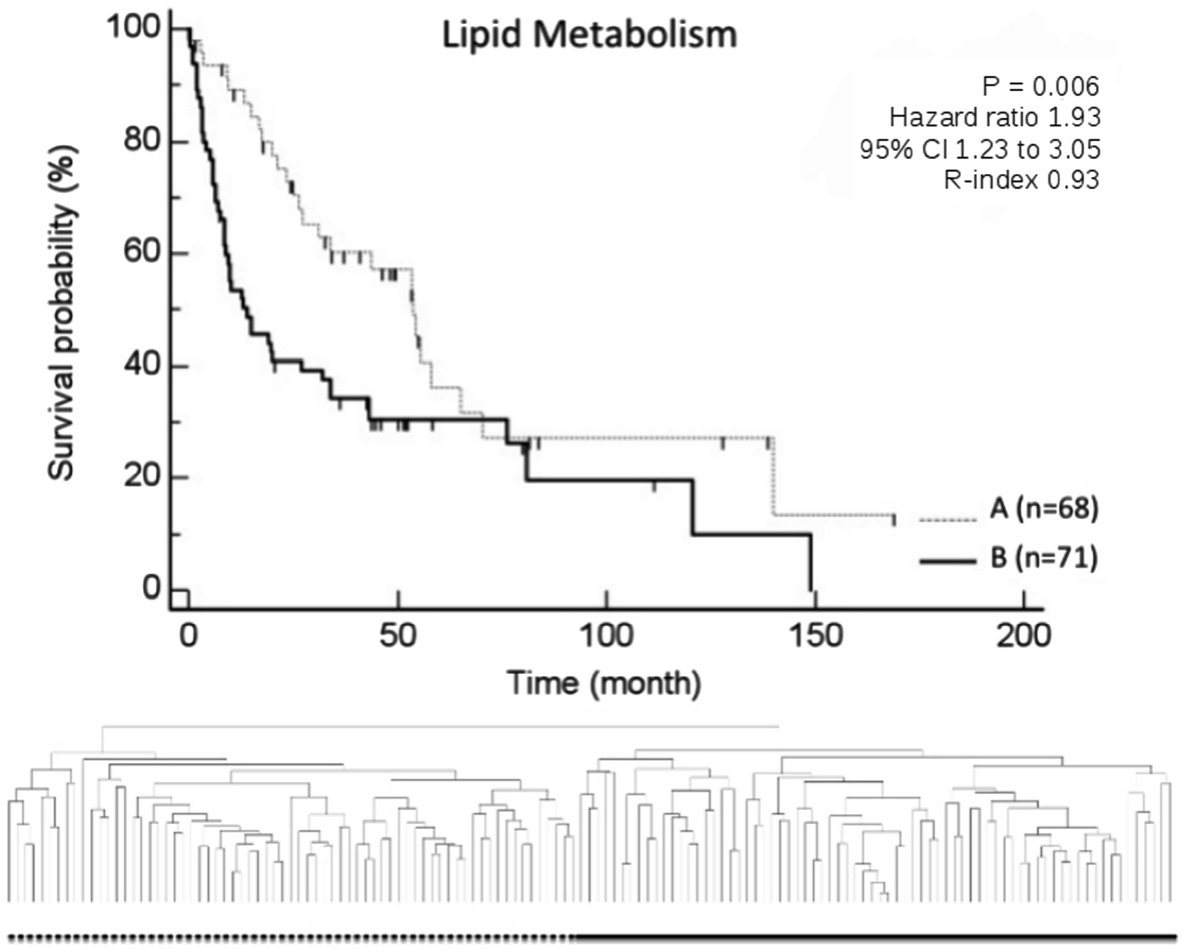
**Besides Carbohydrate metabolism, the lipid metabolism was also demonstrated to be of high relevance.** Based on the expression profile of genes associated with the lipid metabolism, Kaplan Meier estimated survival resulted in two prognostically distinct groups. These subgroups were identified by unsupervised clustering of 139 patients with HCC based on the pathway category “lipid metabolism” (group A contained 68 patients and group B contained 71 patients; p-values were determined by log-rank test).

Since we were able to demonstrate a significant overlap in specific categories of genetic signaling pathways, especially “signaling molecules”, “cell growth and death”, “lipid metabolism” and “carbohydrate metabolism” (Figure 1), we next analyzed the potential biological relevance of these signatures in a data set of 139 human HCC [27,32]. The pathways for “signaling molecules”, “lipid metabolism”, and “carbohydrate metabolism” in particular lacked previous investigation. All four signatures were demonstrated to define prognostically relevant subgroups of HCC by means of unsupervised clustering on the basis of differential regulation of the genes of these specific signatures (Figures 2, 3, 4, 5).

Of all 712 genes associated with signaling pathways of the category “signaling molecules”, 57 were demonstrated to be regulated more than 1.5 fold in at least 20% of data and less than 5% of data missing. Unsupervised clustering of 139 patients with HCC on the basis of these genes resulted in two prognostically diverse subgroups (p = 0.001, adj p-value 0.003, R-index 0.75, indicating agreement between clustering results on 1000 randomly perturbed data sets, where Gaussian noise was added to the log-gene expression measurements) suggesting a biological relevance of this genetic signatures with respect to survival.

Of the 254 genes associated with signaling pathways of the category “cell growth and death”, 33 were demonstrated to be regulated with the same criteria of regulation. Unsupervised clustering of 139 patients with HCC on the basis of these genes also resulted in two prognostically diverse subgroups (p = 0.0005, adj p-value 0.002, R-index 0.8). These findings support an essential role of “cell growth and death” associated genes and signaling pathways with respect to survival.

Most interestingly, we identified for the first time an important role of metabolic signaling pathways in HCC. 343 genes were associated with signaling pathways of the category “lipid metabolism”. Of these, 116 were demonstrated to be regulated more than 1.5 fold in at least 20% of data and less than 5% of data missing. Of 309 genes associated with signaling pathways of the category “carbohydrate metabolism”, 104 were demonstrated to be regulated given the same criteria. Unsupervised clustering of 139 patients with HCC on the basis of both these metabolic categories of signaling pathways resulted in two prognostically distinct subgroups (lipid metabolism: p = 0.006, adj p-value 0.011, R-index 0.93; carbohydrate metabolism: p = 0.013, adj p-value 0.013, R-index 0.99). These data confirmed for the first time a significant role of the comprehensive number of metabolic signaling pathways in HCC with respect to survival.

High cluster reproducibility for all four signatures was demonstrated by an R-index between 0.75 and 0.99 based on 1000 perturbations [34].

## Discussion

In the past, several individual genes were shown to be differentially regulated in liver development and HCC. For example, many of the pathways summarized by Lemaigre [35] to be essential to apoptotic and growths mechanisms during liver development were previously also reported to play essential roles in tumor development [9,12,14,16,20]. In a larger biological context this seems reasonable as multiple general biological mechanisms are common to both liver development and liver cancer, such as cell cycle regulation, tissue growth or regulation of apoptosis. The multiple reports available on individual genes called for a systematic study on commonly differentially regulated genetic patterns relevant to both liver development and HCC. However, such a systematic review on either individual genes or superimposed signaling pathways is still lacking.

In order to gain substantial insight in relevant overlapping biological mechanisms involved in embryonic liver and cancer development, we performed a comprehensive analysis of differentially regulated genetic signaling pathways in both conditions. The identification of genetic networks regulating HCC development and the course of disease are of significant importance as they may not only provide deeper insight into the underlying biology but also point towards novel therapeutic targets. Furthermore, the intersection between liver development and liver growth may aid in identifying driver genes truly involved in these biological mechanisms, and separating them from by-stander genes being differentially regulated in one of these conditions but not essential to the biological mechanisms fundamental to liver development or cancer growths.

Most strikingly, we were able to demonstrate for the first time a substantial overlap between signaling pathways involved in embryonic liver development and liver cancer development. Of the 258 pathways listed in the KEGG signaling pathway database, a total of 112 (43%) pathways were deregulated in at least one malignant cancer array and one developmental array. Among the signaling pathways showing enrichment of differentially regulated genes were several pathways, which we would have anticipated being so, such as signaling molecules. These biological mechanisms pathways were previously demonstrated to be essential to cell cycle, cell division and tissue growth [36,37] and thus the enrichment of these pathways among the differentially regulated genes demonstrated to be relevant to liver development and HCC served as a control for the feasibility of our approach. In contrast, several signaling pathways that were previously reported to be critical to tumor development such as Wnt [9], Jak-Stat [14], MAPK [16], TGF beta [12] and others [18,20] demonstrated an enrichment of differentially regulated genes in only a few individual array experiments but not a significant or high overlap to genetic pathways enriched in embryonic development of the liver. A more detailed view on pathway regulation with respect to the diverse ethnic background of patients in the human data set (78 Caucasian, 61 Chinese) [32] may identify even more enriched pathways.

However, our analysis of overlapping signaling pathways between liver development and HCC development shed light on novel biological aspects of tumor development as these mechanisms demonstrated a high conservation throughout embryonic growths and multiple HCC array experiments.

It was only in the past few years that a significant relevance of metabolic mechanisms in cancer biology was recognized. With respect to carbohydrate metabolism indications came from clinical studies demonstrating that patients with diabetes mellitus have a higher incidence in HCC development and also its presence worsens the prognosis of an existing HCC [38,39]. Similarly the community is beginning to realize that other metabolic changes may contribute to cancer biology. With respect to lipid metabolism almost no experiments have been performed or published so far. The only study touching the subject demonstrated that the risk of HCC in patients with chronic hepatitis C increases in proportion to BMI in a wide range of its values, from underweight to obese [40].

Although from a clinical perspective this link between metabolic disease and cancer must be regarded as being reliable, the underlying molecular mechanisms remain elusive and have only marginally been studied. Our extensive screen for enriched and conserved pathways between liver development and liver cancer pointed towards a significant role of carbohydrate and lipid metabolism in both conditions. These two metabolic pathway categories were among the most enriched signaling pathways among all KEGG curated pathways. Besides speculations about the biological meaning of this broad overlap such as an involvement in stem cell biology (see below), these observervations called for an analysis of their medical relevance for the development and prognosis of HCC. We were able to demonstrate a relevance of both metabolic pathways “lipid metabolism” and “carbohydrate metabolism”. Unsupervised clustering of these patients on the basis of differentially genes enriched in either carbohydrate or lipid metabolism, resulted in a separation of two significantly diverse subgroups showing significantly distinct survival. Thus, we furthermore confirmed a biological significance of our findings with respect to a systems biology view on metabolic pathways. This also defined novel characteristic and prognostically relevant biological mechanisms which would be worth exploring further in future experiments.

Viewing our results in a more general context of cancer development, these results may also shed light onto the upcoming discussion on embryonic stem cells and the state of differentiation in liver cancer.

During recent years these observations have been of renewed interest as they would go along well with a cancer stem cell hypothesis for hepatocellular carcinoma. However, such a stem cell hypothesis is still a matter of debate [41]. Our results showed broadly overlapping genetic mechanisms leading to the biological changes during embryonic liver development and HCC. However, the question whether these signatures were re-activated in previously differentiated cells or whether they represent an early developmental stage during stem/progenitor cell differentiation requires further investigation. In any case, our data support a strong link between embryonic liver and liver cancer development.

## Conclusion

Together, we demonstrated for the first time a significant overlap between genetic signaling pathways, and therefore biological mechanisms, between liver cancer and embryonic liver development using a comprehensive systems biology approach to pathway analysis of genome wide microarray data. The pathway categories with highest overlap in enrichment of regulated genes not only pointed out common biological mechanisms, but were demonstrated to provide novel prognostically relevant genetic signatures in HCC. In particular, metabolic pathways relating to carbohydrate or lipid metabolism had not previously been recognized as having prognostic relevance.

## Material and methods

### Microarray datasets

Murine microarray datasets from experiments studying the development of HCC were retrieved from the GEO database (http: http://www.ncbi.nih.gov/geo/, Table 1). All studies had been performed using the Affymetrix MG 430 2.0 chips ( http://www.affymetrix.com). The GSE13149 dataset provided gene expression profiles during embryonic development at days E11.5, E12.5, E13.5, E14.5, E15.5, E16.5, E17.5, and E18.5 days post conception (dpc) as well as day0, day3, day7, day14, day21 postnatal. These data were compared to gene expression profiles of normal adult mouse liver tissues (18 weeks). In the GSE11201 series gene expression profiles during embryonic development at days E10.5, E11.5, E12.5, E13.5, E14.5, E16.5 days post conception (dpc) is compared with normal adult liver (10 weeks). Finally, gene expression profiles of HCC specimens taken from Mdr-2 knockout mice and Trim24 knockout mice were compared to adjacent liver tissues and normal liver taken from wild type littermates, respectively.

The gene expression data set for studying gene expression in human HCC contained 139 [27,32] 70-mer oligo microarrays consisting of 21 329 genes, which were produced at the Advanced Technology Center at the National Cancer Institute. The dataset includes two ethnic groups (61 Chinese and 78 white) with 73.3% male individuals. The median duration of follow up was 23.4 months and the median age 57. As reference for all microarray experiments pooled RNA from 19 normal livers was used.

**Table 1:** Microarray datasets used in this study

**GEO Identifier**	**Description**	**No. of arrays**	**Microarray platform**	**Reference**
GSE13149	Mouse liver development; time series	26	Affymetrix Mouse Genome 430 2.0 Array	[21]
GSE11201	Mouse liver development; time series	14	Affymetrix Mouse Genome 430 2.0 Array	-
GSE9012	Trim24/TIF1-alpha knockout mouse HCC model; liver samples	10	Affymetrix Mouse Genome 430 2.0 Array	[42]
GSE8642	Mdr-2 knockout mouse HCC model; liver samples	12	Affymetrix Mouse Expression 430A Array	-
GSE1898 / GSE4024	Human HCC; liver samples	139	NCI/ATC Hs-OperonV2	[27,32]

### Microarray data normalisation

All gene expression data for mouse datasets were normalized by computing the RMA (Robust Multichip Average) [43] directly from Affymetrix CEL files of embryonic mouse liver tissues, mouse liver tumor tissues and adult mouse normal liver tissues. The Bioconductor package (R-package) containing the RMA implementation “affy” was installed by accessing the biocLite.R script directly from the Bioconductor website (http: http://www.bioconductor.org/biocLite.R). Normalized data from human HCC microarrays was generated by median over array normalization.

### Selection of differentially regulated genes

Using the FTP annotation files for mouse genome ( ftp://ftp.genome.ad.jp/pub/kegg/genes/organisms/mmu) and human genome ( ftp://ftp.genome.ad.jp/pub/kegg/genes/organisms/hsa) from the KEGG PATHWAY DATABASE ( http://www.genome.jp/kegg/), a list of KEGG associated genes was identified. KEGG-related genes with expression values at least twofold higher or at least twofold lower between human liver tumor and human normal liver samples, mouse tumor liver and normal mouse liver samples, and mouse embryonic (and after birth, developing) liver and adult mouse liver samples were selected as differentially regulated genes.

### KEGG pathway analysis

A functional gene enrichment analysis was performed based on the KEGG listed pathways [29,30]. Whether the percentage of genes with altered expression in a certain pathway differed from the percentage of altered genes not represented in the pathway was tested by Fisher exact test. KEGG pathways with at least two differentially regulated genes and a p-value of < 0.05 were considered “enriched”. As the Fisher Exact tests were only used as a tool to select enriched pathways for further analyses, no adjustment for multiple testing was performed. Since the Lee dataset demonstrated at least two subgroups of patients we investigated the enrichment of signalling pathways also for genes deregulated only in 20% (Lee20), 50% (Lee50), 70% (Lee70) or 80% (Lee80) of patients. Results were confirmed in a second human HCC dataset [33].

### Evaluation of the biological relevance of highly overlapping genetic signaling pathways in human hepatocellular carcinomas

To investigate the prognostic relevance of the individual genetic signaling pathway signatures in HCC, we analyzed a comprehensive data set containing 139 genome wide HCC tissue microarrays [27,32]. Gene names (Additional file 2: Table S6) and identifiers were retrieved from the KEGG database. Initial data analysis was made using the BRB array tools ( http://linus.nci.nih.gov/BRB-ArrayTools.html). Initially, all genes with a percentage of missing data exceeding 5% and fold-change <1.5 were excluded from further analysis. Next, an unsupervised clustering was performed on the basis of the gene list summarizing all genes being differentially regulated within the pathway category to be investigated (Additional file 3: Figure S8 shows related heatmaps). Hierarchical cluster analysis was perfomed using centered correlation and average linkage available in BRB Array Tools. Cluster reproducibility analysis with 1000 perturbations were realized to measure the proportion of pairs of specimens within a cluster (R-index) [34]. This clustering split the human data set into two subgroups A and B. To compare the difference in survival between these subgroups, Kaplan-Meier survival analysis and log-rank test were used. The analysis was performed by using the MedCalc software packages ( http://www.medcalc.be).

## Competing interest

The authors declare no conflict of interest.

## Authors’ contributions

DB designed the study, carried out the data analysis, manuscript preparation. IS carried out the data analysis. MK carried out the data analysis. FS carried out the data analysis. AGA carried out the data analysis and statistical evaluation. AV carried out the statistical evaluation. HB carried out the statistical evaluation. MB carried out the statistical evaluation. TM carried out the data analysis. ST carried out the data generation and participated in the manuscript proof reading. PRG participated in the data discussion and manuscript preparation. AT designed the study, carried out data analysis, and manuscript preparation. All authors read and approved the final manuscript.

## Supplementary Material

Additional file 1 **Table S7.** KEGG Pathway Analysis on a second human HCC of Microarray-Set GSE25097 [33]. Each grey square at the grid intersection between pathway and developmental stage represents a significant enrichment (black circuits) or under re-presentation (white circuits) of differentially regulated genes of this pathway in the analyzed data set. Click here for file

Additional file 2 **Table S1.** Intersection of Genes within Pathways between human HCC Liver samples and mouse samples (GSE13149, murine HCC: Trim24 knockout mice and Mdr2 knockout mice) for the pathway category “Carbohydrate Metabolism”. Table S2: Intersection of Genes within Pathways between human HCC Liver samples and mouse samples (GSE13149, murine HCC: Trim24 knockout mice and Mdr2 knockout mice) for the pathway category “Lipid Metabolism”. Table S3: Intersection of Genes within Pathways between human HCC Liver samples and mouse samples (GSE13149, murine HCC: Trim24 knockout mice and Mdr2 knockout mice) for the pathway category “Cell Growth and Death”. Table S4: Intersection of Genes within Pathways between human HCC Liver samples and mouse samples (GSE13149, murine HCC: Trim24 knockout mice and Mdr2 knockout mice) for the pathway category “Signaling Molecules and Interaction”. Table S5: P-value and observed/expected ratio range for each Pathway. Table S6: Genes used for survival calculation. Click here for file

Additional file 3 **Figure S8.** Corresponding heatmaps to the Kaplan Meier estimated survival (Figures 2, 3, 4, 5). Click here for file
