# Supplementary figures and images for: Genetic signatures shared in embryonic liver development and liver cancer define prognostically relevant subgroups in HCC
Source: Mol Cancer. 2012 Aug 14;11:55. doi: 10.1186/1476-4598-11-55 (PMC3583209; doi:10.1186/1476-4598-11-55)

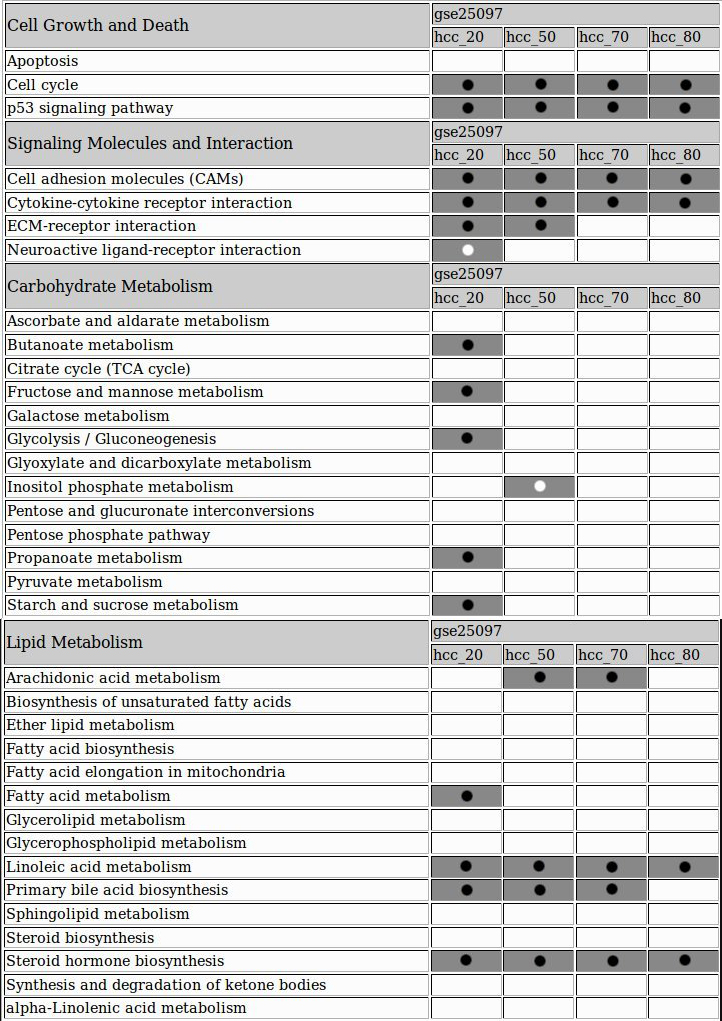

Supplement: Additional file 1 — Table S7. KEGG Pathway Analysis on a second human HCC of Microarray-Set GSE25097 [33]. Each grey square at the grid intersection between pathway and developmental stage represents a significant enrichment (black circuits) or under re-presentation (white circuits) of differentially regulated genes of this pathway in the analyzed data set. [file 1476-4598-11-55-S1.jpeg]

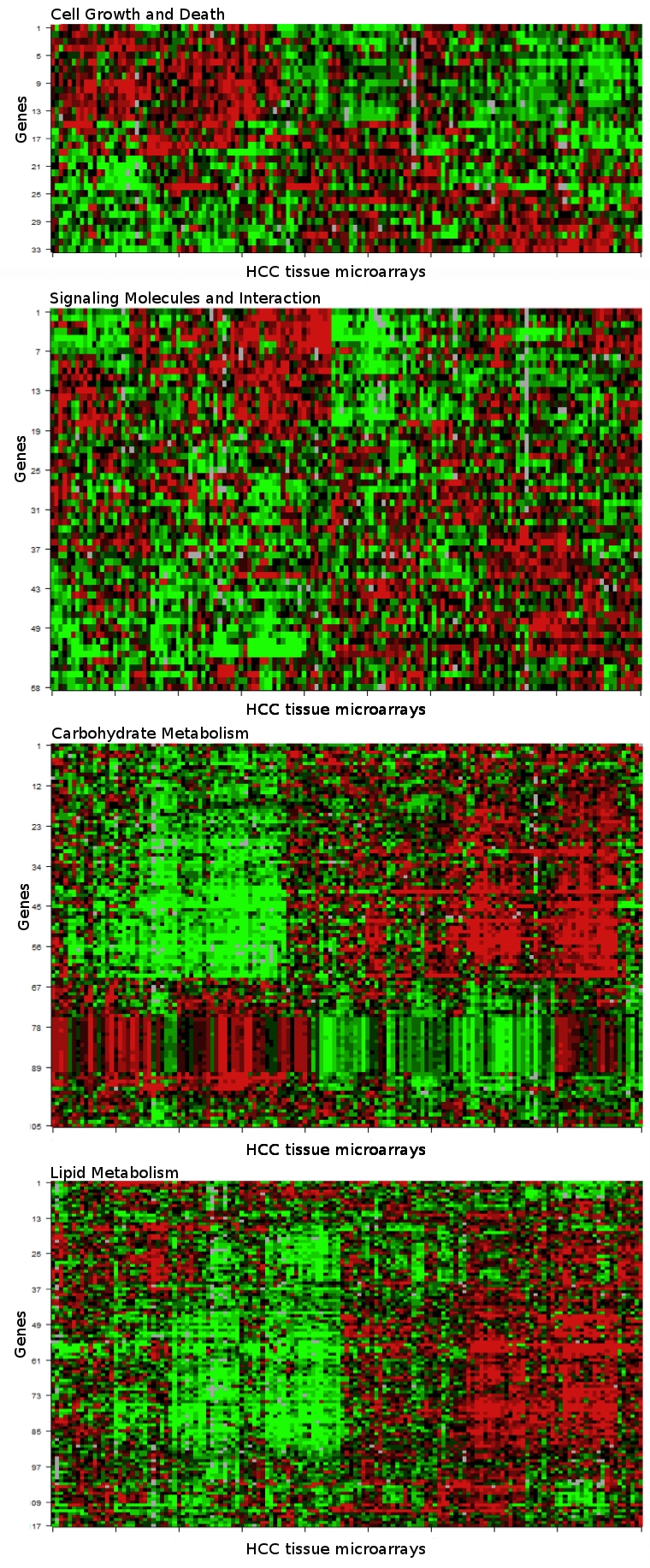

Supplement: Additional file 3 — Figure S8. Corresponding heatmaps to the Kaplan Meier estimated survival (Figures 2, 3, 4, 5). [file 1476-4598-11-55-S3.jpeg]
