# Supplementary material for: Genetic signatures shared in embryonic liver development and liver cancer define prognostically relevant subgroups in HCC
Source: Mol Cancer. 2012 Aug 14;11:55. doi: 10.1186/1476-4598-11-55 (PMC3583209; doi:10.1186/1476-4598-11-55)
Supplement: Additional file 2 — Table S1. Intersection of Genes within Pathways between human HCC Liver samples and mouse samples (GSE13149, murine HCC: Trim24 knockout mice and Mdr2 knockout mice) for the pathway category “Carbohydrate Metabolism”. Table S2: Intersection of Genes within Pathways between human HCC Liver samples and mouse samples (GSE13149, murine HCC: Trim24 knockout mice and Mdr2 knockout mice) for the pathway category “Lipid Metabolism”. Table S3: Intersection of Genes within Pathways between human HCC Liver samples and mouse samples (GSE13149, murine HCC: Trim24 knockout mice and Mdr2 knockout mice) for the pathway category “Cell Growth and Death”. Table S4: Intersection of Genes within Pathways between human HCC Liver samples and mouse samples (GSE13149, murine HCC: Trim24 knockout mice and Mdr2 knockout mice) for the pathway category “Signaling Molecules and Interaction”. Table S5: P-value and observed/expected ratio range for each Pathway. Table S6: Genes used for survival calculation. [file 1476-4598-11-55-S2.doc]

S1: Intersection of Genes within Pathways between human HCC Liver samples and mouse samples (GSE13149, murine HCC: Trim24 knockout mice and Mdr2 knockout mice) for the pathway category “Carbohydrate Metabolism”.

S2: Intersection of Genes within Pathways between human HCC Liver samples and mouse samples (GSE13149, murine HCC: Trim24 knockout mice and Mdr2 knockout mice) for the pathway category “Lipid Metabolism”.

S3: Intersection of Genes within Pathways between human HCC Liver samples and mouse samples (GSE13149, murine HCC: Trim24 knockout mice and Mdr2 knockout mice) for the pathway category “Cell Growth and Death”.

S4: Intersection of Genes within Pathways between human HCC Liver samples and mouse samples (GSE13149, murine HCC: Trim24 knockout mice and Mdr2 knockout mice) for the pathway category “Signaling Molecules and Interaction”.

S5: P-value and observed/expected ratio range for each Pathway.

S6: Genes used for survival calculation.
